# Plasma brain‐derived tau correlates with cerebral infarct volume

**DOI:** 10.1111/joim.20041

**Published:** 2024-12-05

**Authors:** Fernando Gonzalez‐Ortiz, Lukas Holmegaard, Björn Andersson, Cecilia Brännmark, Christian Blomstrand, Henrik Zetterberg, Katarina Jood, Kaj Blennow, Christina Jern, Tara M. Stanne

**Affiliations:** ^1^ Department of Psychiatry and Neurochemistry Institute of Neuroscience and Physiology The Sahlgrenska Academy, University of Gothenburg Mölndal Sweden; ^2^ Region Västra Götaland, Clinical Neurochemistry Laboratory Sahlgrenska University Hospital Mölndal Sweden; ^3^ Department of Clinical Neuroscience, Institute of Neuroscience and Physiology, The Sahlgrenska Academy University of Gothenburg Gothenburg Sweden; ^4^ Region Västra Götaland Department of Neurology Sahlgrenska University Hospital Gothenburg Sweden; ^5^ Bioinformatics and Data Center The Sahlgrenska Academy University of Gothenburg Gothenburg Sweden; ^6^ Department of Laboratory Medicine, Institute of Biomedicine The Sahlgrenska Academy University of Gothenburg Gothenburg Sweden; ^7^ Region Västra Götaland Department of Medicine, Geriatrics and Emergency Medicine Sahlgrenska University Hospital Östra Hospital Gothenburg Sweden; ^8^ Department of Neurodegenerative Disease UCL Institute of Neurology Queen Square London UK; ^9^ UK Dementia Research Institute at UCL London UK; ^10^ Hong Kong Center for Neurodegenerative Diseases Clear Water Bay Hong Kong China; ^11^ Wisconsin Alzheimer's Disease Research Center University of Wisconsin School of Medicine and Public Health University of Wisconsin‐Madison Madison Wisconsin USA; ^12^ Paris Brain Institute ICM Pitié‐Salpêtrière Hospital Sorbonne University Paris France; ^13^ Neurodegenerative Disorder Research Center Division of Life Sciences and Medicine Department of Neurology Institute on Aging and Brain Disorders University of Science and Technology of China and First Affiliated Hospital of USTC Hefei China; ^14^ Region Västra Götaland Department of Clinical Genetics and Genomics Sahlgrenska University Hospital Gothenburg Sweden

**Keywords:** blood biomarkers, brain‐derived tau, infarct volume, stroke, stroke severity

## Abstract

**Background:**

A blood‐based biomarker that accurately reflects neuronal injury in acute ischemic stroke could be an easily accessible and cost‐effective complement to clinical and radiological evaluation. Here, we investigate whether plasma levels of the novel biomarker brain‐derived tau (BD‐tau) reflect cerebral infarct volumes and whether BD‐tau can improve clinical outcome prediction.

**Methods:**

The present study included 713 consecutive cases from two different hospital‐based cohorts, *the* *Sahlgrenska Academy Study on Ischemic Stroke* (*SAHLSIS*) and *SAHLSIS phase 2* (*SAHLSIS2*). Acute stroke severity was determined by the Scandinavian Stroke Scale converted to the National Institutes of Health stroke scale (NIHSS) in *SAHLSIS* and by the NIHSS in *SAHLSIS2*. All participants were assessed for functional outcome 3 months after stroke by the modified Rankin Scale, and 254 participants in *SAHLSIS* had quantitative neuroimaging available.

**Findings:**

Plasma BD‐tau concentrations and cerebral infarct volumes were highly correlated (*ρ* 0.72, *p* < 0.001). BD‐tau improved the prognostic accuracy of suffering an unfavorable outcome over age and stroke severity in the whole cohort. However, the gain in predictive power was dependent on stroke severity and infarct location. The largest improvement was observed for mild ischemic strokes (NIHSS <5; area under the curve [AUC] = 0.73 for age + NIHSS versus AUC = 0.84 with addition of BD‐tau; DeLong *p* 0.02), posterior circulation stroke (AUC = 0.75 vs. AUC = 0.84; DeLong *p* 0.06) and more specifically for infarcts in the brainstem/cerebellum (AUC = 0.74 vs. 0.87; DeLong *p* 0.009).

**Conclusion:**

Plasma BD‐tau can provide information on the extent of acute neuronal damage in ischemic stroke and adds prognostic value for outcome, especially for mild and posterior circulation strokes.

## Graphical abstract

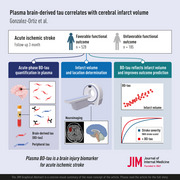

## Introduction

Stroke continues to be a major cause of disability and mortality globally, and incidence among people younger than 70 years of age is increasing [1]. Despite advances in diagnostic modalities and treatments, accurate prognostication remains a significant challenge. As accurate prognostication is imperative to optimize therapeutic decisions and to inform healthcare teams, patients, and caregivers, an enhanced understanding of predictive variables that can improve prognostic accuracy is warranted.

The National Institutes of Health Stroke Scale (NIHSS) is a neurological deficit rating scale originally intended to define selection criteria in randomized controlled trials (RCTs) of ischemic stroke [2]. It is now also widely used in the clinic, and it is the strongest known predictor of functional outcome after ischemic stroke followed by age [3]. However, the predictive accuracy of the NIHSS score differs between anterior versus posterior circulation strokes [4], and it fails to capture some important deficits that can affect functional outcomes in cases presenting with mild symptoms [5].

Although assessments of cerebral infarct volumes have proven to enhance outcome prediction [6, 7], this information is not readily available for all ischemic stroke patients. Non‐contrast computed tomography (CT) of the brain is the primary neuroimaging modality used in the acute clinical stroke setting, and it can differentiate ischemic from hemorrhagic stroke. However, with regards to ischemic stroke, CT imaging is unreliable for detecting small cerebral infarcts and for predicting final infarct volume compared to magnetic resonance imaging (MRI) [8, 9]. Posterior circulation infarcts are particularly difficult to detect on CT‐based neuroimaging [10]. Although MRI has relatively higher sensitivity and accuracy in detecting infarcts compared to CT, it is not always feasible due to restricted availability, contraindications, and cost [9, 11, 12].

Given the limitations of early neuroimaging and the NIHSS for prognostication in current clinical practice, there remains a need for improved prognostic measures of ischemic stroke. Here, a blood‐based biomarker capable of accurately tracking acute neuronal injury after an ischemic stroke could have potential clinical utility as an objective tool to complement clinical evaluations and early neuroimaging. Unlike for myocardial infarction, where cardiac troponins measured in the blood specifically reflect the extent of heart muscle damage and are now routinely implemented in clinical practice as a complement to electrocardiogram and clinical symptom assessment [13, 14], no blood‐based biomarker accurately reflecting acute neuronal damage is currently available for ischemic stroke [15]. Due to the activation of cell death pathways [16] and the disruption of blood‐brain barrier (BBB) integrity during cerebral ischemia [17], we hypothesize that an analogous blood‐based brain injury biomarker exists for acute ischemic stroke.

Measurements of non‐phosphorylated tau, or “total tau” (T‐tau) in cerebrospinal fluid (CSF) is an established biomarker of neurodegeneration intensity in Alzheimer's disease (AD) [18], and increased concentrations of T‐tau in blood have also been reported in different acute neurological conditions including acute ischemic stroke [19, 20, 21, 22, 23, 24]. However, T‐tau has limited clinical utility as an AD biomarker in blood [24], due to tau being expressed in different locations outside the brain, for example, the peripheral nervous system and heart tissue [25, 26]. This makes standard blood‐based T‐tau assays unreliable for the determination of the extent of central nervous system (CNS) insults as the T‐tau signal in blood represents a combination of peripheral‐derived tau (80%) and brain‐derived tau (BD‐tau) (20%) [18]. Considering this, an assay for circulating levels of BD‐tau has been developed, and this novel assay takes advantage of the architectural difference between peripheral and CNS tau to quantify tau predominantly of brain origin, hence its name [18]. In contrast to T‐tau, blood levels of BD‐tau were demonstrated to be highly correlated with tau levels in CSF in neurodegenerative conditions and reflect the extent of neuronal death [18, 27, 28]. Further, blood levels of BD‐tau were established to be elevated in acute ischemic stroke patients compared to healthy controls [29], a likely reflection of neuronal death and BBB breakdown following cerebral ischemia [17]. Moreover, increased blood BD‐tau concentrations were associated with unfavorable outcomes after traumatic brain injury [30] and ischemic stroke [29, 31], outperforming other brain injury markers such as neurofilament light (NfL) and glial acidic fibrillar protein (GFAP) at tracking CNS insults [30, 31, 32].

Based on these findings and BD‐tau's CNS specificity, we hypothesize that acute‐phase plasma BD‐tau should be capable of reflecting infarct volume across different brain locations in ischemic stroke and may add valuable information to clinical stroke severity scales and CT neuroimaging, especially for minor and posterior circulation ischemic strokes.

## Methods

### Study population

Participants were from the hospital‐based observational cohort study, the *Sahlgrenska Academy Study on Ischemic Stroke* (*SAHLSIS*) and *SAHLSIS phase 2* (*SAHLSIS2*). Both have been described previously [33, 34], and details are given in the Supplementary Methods. In brief, patients with first‐ever or recurrent acute ischemic stroke aged 18–69 years were recruited to *SAHLSIS* between 1998 and 2003, and adults of all ages were recruited 2015–2020 to *SAHLSIS2*. For the present study, cases from *SAHLSIS* with remaining aliquoted plasma and a complete set of relevant clinical data (i.e., acute NIHSS score and 3‐month modified Rankin Scale (mRS) score) were selected (*n* = 454), for *SAHLSIS2* consecutive patients who did not undergo thrombectomy with a complete set of relevant clinical data were selected (*n* = 259). Recruitment to *SAHLSIS* took place before treatment with thrombectomy was part of the clinical routine. Index strokes were categorized using the Oxfordshire Community Stroke Project (OCSP) classification [35] into total or partial anterior circulation infarct (TACI + PACI), posterior circulation infarct (POCI), and lacunar infarct. In *SAHLSIS n* = 3 and in *SAHLSIS2 n* = 33 cases were not classified by the OCSP. All participants were assessed for functional outcomes 3 months after the stroke by the mRS. No participant was lost to follow‐up.

### Stroke severity

In *SAHLSIS*, stroke severity, that is, neurological deficit, was measured using the Scandinavian Stroke Scale (SSS), which has been demonstrated to have comparable performance to the NIHSS in predicting death or dependence after stroke [36]. The score reflecting the highest neurological deficit within 7 days of hospital admission was converted to the NIHSS score using an established algorithm [37]. In *SAHLSIS2*, stroke severity was defined using the NIHSS score either at admission for patients who did not receive intravenous thrombolysis or 24 h after thrombolysis treatment.

### Neuroimaging and infarct volume measurement

All cases underwent CT of the brain in the acute phase according to clinical routine. A subgroup of *SAHLSIS* (*n* = 254) underwent MRI of the brain with infarct volume measured as described [38]. In brief, proton density, T1‐weighted, and T2‐weighted sequences were acquired. In some cases, depending on the clinical context and the scanner available, additional sequences were obtained. All MRI examinations in this subgroup were reviewed and assessed by one neuroradiologist (C. Jensen). Quantitative measurements of infarct volumes were then performed by one stroke neurologist (L.H.). Focal lesions were manually delineated, and volumes were calculated using 3D Slicer version 3.10.2 (National Alliance for Medical Image Computing, USA). No visible infarct was detected for *n* = 21 individuals. The remaining infarcts were categorized into MRI‐based vascular territory (right or left anterior and posterior circulation) or MRI‐based anatomical location (right or left hemisphere and brainstem or cerebellum). As the focus of our study was to investigate the association between infarct volumes and the concentration of BD‐Tau, infarct volumes were not adjusted for total brain volume. For the remaining study participants, infarct locations were defined based on symptoms and, when possible, also based on radiology reports from CT/MRI of the brain into the anatomical location categories: right or left hemisphere and brainstem or cerebellum.

### Blood sampling and protein measurement

Peripheral venous blood was drawn after an overnight fast at inclusion (median 4 [IQR 3–6] and 2 [IQR 2–4] days after index stroke in *SAHLSIS* and *SAHLSIS2*, respectively). EDTA plasma was aliquoted and frozen, and BD‐tau measurements were performed following thawing. All samples from each cohort were analyzed in one round of experiments using one batch of reagents on the Simoa HD‐X platform (Quanterix) at the Clinical Neurochemistry Laboratory, Sahlgrenska University Hospital, Mölndal, Sweden, as described [18]. Dyed beads were used in *SAHLSIS*, and carboxylated beads were used in *SAHLSIS2*. Quality control samples were analyzed in duplicates at the start and at the end of each plate to assess precision. The inter‐assay coefficient of variation (CV) was 10.9% and 8.5%, and the intra‐assay CV was 6.1% and 3.8% for *SAHLSIS* and *SAHLSIS2*, respectively.

### Statistical analysis

Spearman's rank test evaluated the correlation among BD‐tau, infarct volumes, and the NIHSS score in *SAHLSIS*. In the combined *SAHLSIS* and *SAHLSIS2* cohort, we used binary logistic regression to investigate associations among Ln‐transformed BD‐tau, age, and NIHSS score with unfavorable 3‐month outcome (mRS score 3–6) as described [29]. We then used receiver operating characteristics curve analyses to assess the accuracy of each model for functional outcome prediction. Sensitivities and specificities (Youden index) as well as positive and negative prediction values were computed, and the resulting area under the curve (AUC) for models with and without LnBD‐tau were compared using a DeLong test. The procedures described above were repeated stratified by vascular territory (anterior vs. posterior) and by anatomical location (right or left hemisphere vs. brainstem or cerebellum) as well as by stroke severity (mild stroke defined as NIHSS score 0–4 vs. more severe stroke defined as NIHSS score ≥5). Finally, as stroke severity in *SAHLSIS* was based on NIHSS converted from SSS, a sensitivity analysis using only *SAHLSIS2* was performed. Two‐tailed *p *< 0.05 was considered significant.

### Standard protocol approvals

Written informed consent was obtained by all participants or next‐of‐kin according to the Declaration of Helsinki. *SAHLSIS* was approved by the Regional Ethics Review Board in Gothenburg, Sweden (469‐99, T553‐03, and 413‐04, T665‐07) and *SAHLSIS2* by the Regional Ethics Review Board in Gothenburg (823‐13, T1110‐16) and the Swedish Ethics Review Authority (2022‐00597‐02).

### Data availability

Anonymized data will be shared upon reasonable request, provided the data transfer agrees with EU legislation on the general data protection regulation and with decisions by the Ethical Review Board of Sweden and the University of Gothenburg, the latter which should be regulated in a data transfer agreement.

## Results

The baseline characteristics from the *SAHLSIS* subgroup with quantitative neuroimaging and for the *SAHLSIS* and *SAHLSIS2* cohorts are shown in Table 1.

**Table 1:** Baseline characteristics for ischemic stroke cases in the Sahlgrenska Academy Study on Ischemic Stroke (SAHLSIS) and SAHLSIS phase 2 (SAHLSIS2).

	*SAHLSIS* MRI subgroup	*SAHLSIS*	*SAHLSIS2*
Ischemic stroke cases, *n*	254	454	259
Age, median [IQR], years	55 [47–62]	58 [52–64]	67 [57–79]
Male sex, *n* (%)	168 (66)	303 (67)	164 (63)
Hypertension, *n* (%)	139 (55)	270 (60)	116 (45)
Diabetes mellitus, *n* (%)	44 (17)	87 (19)	34 (13)
Smoker, *n* (%)	100 (39)	178 (39)	29 (11)
Thrombolysis, *n* (%)	0 (0)	0 (0)	56 (22)
Scandinavian Stroke Scale (SSS) score, median [IQR]	54 [45–56]	54 [45–56]	–
NIH Stroke Scale (NIHSS) score, median [IQR]	2 [2–6]^a^	2 [1–6]^a^	2 [1–6]
Mild stroke (NIHSS 0–4), *n*	170	306	171
More severe stroke (NIHSS 5–42), *n*	84	148	88
Day of blood draw, median [IQR]	4 [3–6]	4 [3–6]	2 [2–4]
Plasma BD‐tau, median [IQR], pg/mL	5.3 [3.8–14.7]	5.4 [3.8–15.2]	14.7 [7.3–35.6]
modified Rankin Scale (0–2 vs. 3–6), *n*	189 vs. 54	351 vs. 103	177 vs. 82
**MRI‐based vascular territory, *n* (%)**			
Right anterior circulation	49 (19)	–	–
Left anterior circulation	75 (29)	–	–
Posterior circulation	83 (33)	–	–
Both right + left anterior	15 (6)	–	–
Both anterior + posterior	11 (4)	–	–
**MRI‐based anatomical location,** *n* **(%)**			
Right hemisphere	60 (24)	–	–
Left hemisphere	89 (35)	–	–
Brainstem or cerebellum	38 (15)	–	–
Both right + left hemisphere	33 (13)	–	–
Both right or left + brainstem or cerebellum	13 (5)	–	–
**OCSP classification,** *n* **(%)**			
TACI, total anterior circulation infarct	18 (7)	38 (8)	25 (11)
PACI, partial anterior circulation infarct	81 (32)	138 (30)	93 (41)
POCI, posterior circulation infarct	75 (30)	117 (26)	59 (26)
LACI, lacunar infarct	78 (31)	158 (35)	49 (22)
**Symptoms and neuroimaging‐based anatomical location,** *n* **(%)**			
Right hemisphere	–	152 (34)	87 (43)
Left hemisphere	–	213 (47)	74 (37)
Brainstem or cerebellum	–	83 (18)	40 (20)
More than one	–	6 (1)	0 (0)

Abbreviations: MRI, magnetic resonance imaging; NHSS, National Institutes of Health stroke scale; OCSP, Oxfordshire Community Stroke Project.

^a^ Converted from SSS score.

### SAHLSIS MRI subgroup

**Fig. 1:**
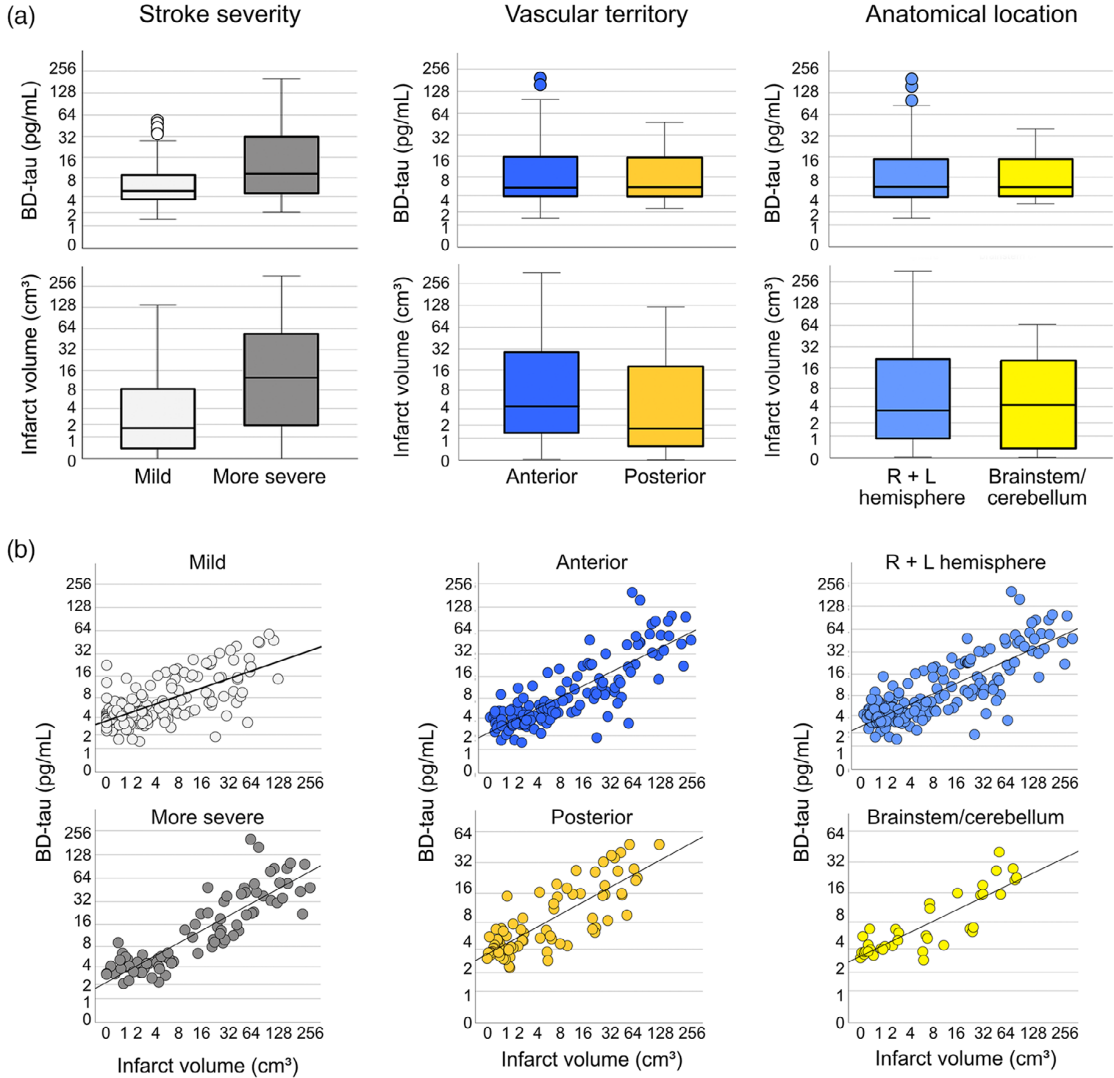
**Plasma brain‐derived tau (BD‐tau) concentrations are correlated with infarct volume in acute ischemic stroke**. (a) Box plots of BD‐tau concentrations (top) and infarct volumes (bottom) stratified by stroke severity, magnetic resonance imaging (MRI)‐based vascular territory, and anatomical location. (b) Scatter plots of BD‐tau versus infarct volumes stratified by stroke severity and infarct location.

MRI was performed at a median of 7 days after index stroke (IQR 4–75 days), and blood was sampled at a median of 3 days (IQR −1 to 70 days] prior to MRI. The median infarct volume was 2.5 cm^3^ [IQR 0.6–19.7 cm^3^], and the median BD‐tau concentration was 5.3 pg/mL [3.8–14.7 pg/mL] (Fig. 1a). BD‐tau and infarct volumes were strongly correlated in all ischemic stroke (*ρ* 0.72, *p* 9 × 10^−42^), and in analyses stratified by stroke severity and infarct location (Fig. 1 and Table 2). In exploratory analyses stratified by median day of blood draw or median day of MRI, BD‐tau concentrations were similar, as were correlations between BD‐tau and infarct volume (see Section S2 for details).

**Table 2:** Spearman's rank correlation coefficients (ρ) for BD‐tau, infarct volume and National Institutes of Health Stroke Scale (NIHSS) score in the Sahlgrenska Academy Study on Ischemic Stroke (SAHLSIS) magnetic resonance imaging (MRI) subgroup for all Ischemic stroke and stratified based on stroke severity, MRI‐based vascular territory, and anatomical location.

		BD‐tau and infarct volume		Infarct volume and NIHSS		BD‐tau and NIHSS	
	*N*	*ρ*	*p*	*ρ*	*p*	*ρ*	*p*
All ischemic stroke	254	0.72	9E − 42	0.44	3E − 13	0.29	3E − 06
Minor stroke (NIHSS <5)	170	0.59	3E − 17	0.28	0.001	0.091	n.s.
More severe stroke (NIHSS ≥5)	84	0.88	4E − 28	0.48	5E − 06	0.41	1E − 04
Anterior circulation	139^a^	0.77	8E − 29	0.51	1E − 10	0.46	9E−09
Posterior circulation	83^a^	0.77	2E − 17	0.20	n.s.	−0.01	n.s.
Right or left hemisphere	182^b^	0.74	4E − 32	0.47	2E − 11	0.34	2E − 06
Brainstem or cerebellum	38^b^	0.84	6E − 11	0.25	n.s.	0.14	n.s.

^a^ No visible infarct detected upon MRI, *n* = 21; and infarct in both anterior and posterior circulation, *n* = 11.

^b^ No visible infarct detected upon MRI, *n* = 21; and infarct in both right or left hemisphere and brainstem or cerebellum, *n* = 13.

The NIHSS score (converted from the SSS score) was moderately correlated to infarct volume and to BD‐tau in more severe strokes, infarcts in the anterior circulation, and in left and right hemisphere strokes, whereas it displayed a weak or no correlation to infarct volume and BD‐tau in minor strokes, posterior circulation strokes, or brainstem or cerebellar strokes (Table 2).

### SAHLSIS and SAHLSIS2

As shown in Table 3, in the combined *SAHLSIS* and *SAHLSIS2* cohort, BD‐tau demonstrated a good prognostic accuracy of functional outcome (AUC = 0.75), and it improved the prognostic capability significantly over age and the NIHSS score, the two strongest known predictors of functional outcome (AUC = 0.85 for age + NIHSS vs. AUC = 0.87 with addition of BD‐tau; DeLong *p* 0.02). When stratified for initial stroke severity, BD‐tau improved the discriminative accuracy for predicting unfavorable functional outcome in both mild strokes (AUC = 0.78 for age + NIHSS vs. AUC = 0.83 with the addition of BD‐tau) and more severe strokes (AUC = 0.73 for age + NIHSS vs. AUC = 0.76 + BD‐tau), though the difference was significant for mild strokes only (mild stroke, DeLong *p* 0.02).

**Table 3:** Discriminative accuracy of BD‐tau and clinical variables for predicting unfavorable functional outcome in the combined Sahlgrenska Academy Study on Ischemic Stroke (SAHLSIS) and SAHLSIS phase 2 (SAHLSIS2) cohort using receiver operating characteristic (ROC) curve analysis.

Model	AUC (95% CI)	*p*	Specificity and sensitivity	Positive, negative predictive values
**All Ischemic stroke**				
Ln‐BD‐tau	0.75 (0.70–0.79)	7E − 23	0.73, 0.69	0.47, 0.87
NIHSS	0.81 (0.77–0.85)	3E − 35	0.75, 0.77	0.52, 0.90
NIHSS + age	0.85 (0.82–0.89)	6E − 45	0.79, 0.84	0.58, 0.93
NIHSS + age + LnBD‐tau	0.87 (0.84–0.90)^a^	2E − 50	0.83, 0.81	0.62, 0.93
**Mild stroke (NIHSS < 5)**				
Ln‐BD‐tau	0.79 (0.72–0.86)	2E − 11	0.75, 0.76	0.29, 0.96
NIHSS	0.65 (0.56–0.73)	2E − 04	0.56, 0.74	0.18, 0.94
NIHSS + age	0.78 (0.71–0.86)	3E − 11	0.89, 0.61	0.43, 0.95
NIHSS + age + LnBD‐tau	0.83 (0.76–0.90)^a^	8E − 15	0.85, 0.78	0.41, 0.97
**More severe stroke (NIHSS ≥ 5)**				
Ln‐BD‐tau	0.70 (0.63–0.76)	1E − 07	0.67, 0.66	0.71, 0.62
NIHSS	0.69 (0.62–0.76)	3E − 07	0.55, 0.75	0.67, 0.65
NIHSS + age	0.73 (0.63–0.76)	2E − 09	0.66, 0.71	0.72, 0.66
NIHSS + age + LnBD‐tau	0.76 (0.70–0.82)	2E − 12	0.69, 0.71	0.74, 0.67
**Anterior circulation (TACI+PACI)**				
Ln‐BD‐tau	0.72 (0.66–0.78)	3E − 10	0.62, 0.75	0.56, 0.79
NIHSS	0.80 (0.75–0.86)	4E − 18	0.72, 0.78	0.65, 0.83
NIHSS + age	0.85 (0.81–0.90)	1E − 23	0.71, 0.89	0.67, 0.91
NIHSS + age + LnBD‐tau	0.86 (0.82–0.90)	1E − 24	0.81, 0.82	0.74, 0.87
**Posterior circulation (POCI)**				
Ln‐BD‐tau	0.75 (0.64–0.86)	1E − 04	0.66, 0.84	0.29, 0.96
NIHSS	0.73 (0.61–0.85)	3E − 04	0.78, 0.62	0.33, 0.93
NIHSS + age	0.75 (0.62–0.87)	1E − 04	0.85, 0.62	0.42, 0.93
NIHSS + age + LnBD‐tau	0.84 (0.76–0.92)^b^	9E − 08	0.79, 0.79	0.40, 0.96
**Right + left hemisphere**				
Ln‐BD‐tau	0.74 (0.70–0.79)	3E − 18	0.73, 0.68	0.50, 0.85
NIHSS	0.84 (0.80–0.88)	1E − 33	0.74, 0.82	0.56, 0.91
NIHSS + age	0.87 (0.84–0.91)	7E − 40	0.81, 0.83	0.64, 0.92
NIHSS + age + LnBD‐tau	0.89 (0.85–0.92)	7E − 43	0.77, 0.88	0.60, 0.94
**Brainstem + cerebellum**				
Ln‐BD‐tau	0.78 (0.66–0.90)	4E − 04	0.72, 0.82	0.32, 0.96
NIHSS	0.65 (0.47–0.82)	0.06	0.92, 0.44	0.47, 0.91
NIHSS + age	0.74 (0.58–0.89)	0.002	0.77, 0.69	0.32, 0.94
NIHSS + age + LnBD‐tau	0.87 (0.78–0.96)^c^	2E − 06	0.78, 0.88	0.40, 0.97

Abbreviations: AUC, area under the ROC curve; NIHSS, National Institutes of Health stroke scale; PACI, partial anterior circulation infarct; POCI, Posterior circulation infarct; TACI, total anterior circulation infarct.

^a^ DeLong *p*‐value 0.02 compared to NIHSS + age.

^b^ DeLong *p*‐value 0.06 compared to NIHSS + age.

^c^ DeLong *p*‐value 0.009 compared to NIHSS + age.

**Fig. 2:**
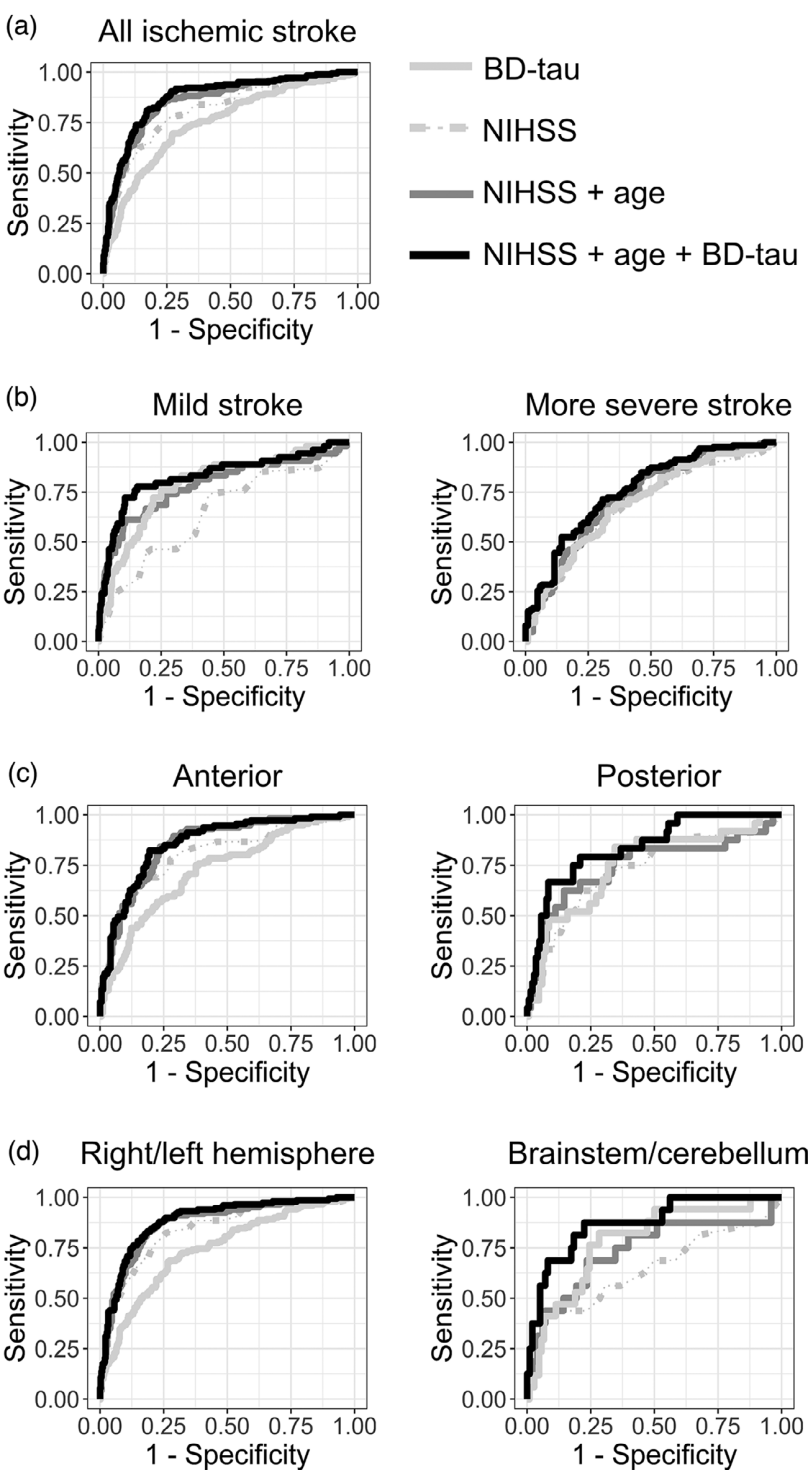
**Receiver operating characteristic curves for accuracy of brain‐derived tau (BD‐tau) and clinical variables for predicting unfavorable outcome**. Analyses were performed in the combined Sahlgrenska Academy Study on Ischemic Stroke (SAHLSIS) + SAHLSIS phase 2 (SAHLSIS2) cohort (a) for all ischemic stroke and stratified based on; (b) clinical stroke severity (i.e., NIHSS < or ≥5); (c) Oxfordshire Community Stroke Project (OCSP) classifications (i.e., vascular territory); and (d) by symptoms‐ and neuroimaging‐based anatomical location. Table 3 provides detailed receiver operating characteristics curve (ROC) analysis.

In analyses stratified by infarct location according to the OCSP classification, BD‐tau had the largest improvement over age and NIHSS in POCIs (AUC = 0.75 age + NIHSS vs. AUC = 0.84 + BD‐tau, DeLong *p* 0.06; Fig. 2) compared to anterior circulation infarcts (AUC = 0.85 age + NIHSS vs. AUC = 0.86 + BD‐tau). Similarly, in analyses stratified by symptoms or neuroimaging‐based anatomical location, the improvement in prognostic accuracy by BD‐tau over age and NIHSS was largest for brainstem or cerebellar infarcts (AUC = 0.74 for age + NIHSS vs. AUC = 0.87 + BD‐tau; DeLong *p* 0.009; Fig. 2). Results from binary logistic regression analyses supporting these AUC analyses are available in Section S2 and Table S1.

It is noteworthy that the prognostic accuracy of BD‐tau alone was superior to the NIHSS for mild strokes (AUC = 0.79 for BD‐tau vs. AUC = 0.65 for NIHSS; DeLong *p* 0.015). BD‐tau had equivalent prognostic accuracy as the NIHSS and age for mild strokes, POCIs, and infarcts in the brainstem or cerebellum (Fig. 2, Table 3).

In a sensitivity analysis using only *SAHLSIS2*, the results for all ischemic stroke and location stratification were consistent with those of the combined cohort (i.e., all ischemic stroke, AUC = 0.82 for age + NIHSS vs. AUC = 0.84 with the addition of BD‐tau; posterior circulation strokes AUC = 0.71 vs. 0.76; and brainstem or cerebellar infarcts AUC = 0.73 vs. 0.80). In exploratory analyses, BD‐tau concentrations were not correlated with the day of blood draw, and there was no significant difference observed in BD‐tau concentrations in first‐ever versus recurrent stroke (see Section S2 for details).

## Discussion

Here, we report that plasma concentrations of BD‐tau are strongly correlated to infarct volume in ischemic stroke patients. Our findings corroborate the CNS origin of BD‐tau and suggest that increased levels of plasma BD‐tau in stroke are indicative of the extent of the acute neuronal injury following acute cerebral ischemia. Additionally, we demonstrate that BD‐tau improves the discriminative accuracy for predicting unfavorable functional outcomes over age and the NIHSS score.

Acute ischemic stroke patients presenting with mild neurological deficits constitute approximately two‐thirds of all strokes, though the definition of mild ischemic stroke is variable [5, 39]. Despite having mild symptoms, these patients present a clinical challenge given their widely variable outcomes and controversy surrounding treatments [39]. Previous studies have shown that the NIHSS score fails to capture some important deficits that can affect functional outcomes in mild ischemic strokes [5]. Here, we found that BD‐tau significantly improved prognostic accuracy over age and the NIHSS score for mild strokes, and on its own, BD‐tau was superior to the NIHSS score. This is despite the relatively lower correlation between BD‐tau and infarct volume in mild strokes compared to more severe strokes. Speculatively, this may reflect the difficulty in accurately ascertaining the volume of small infarcts on neuroimaging and/or that small infarcts may have a more variable impact on outcome depending on the location.

Posterior circulation ischemic stroke accounts for 20%–30% of all ischemic strokes, and the most common regions involved include the brainstem, cerebellum, occipital lobe, and medial temporal lobe [10]. It is well known that the NIHSS score is weighted more toward anterior circulation symptoms and often underestimates clinical severity in patients with infarcts in the posterior circulation [2, 4, 10]. It has previously been reported that infarct volumes measured using diffusion‐weighted MRI or chronic infarct volumes measured by T2‐weighted MRI correlate with the NIHSS score in patients with anterior circulation infarcts [40, 41] but not in patients with infarcts in the posterior circulation [6], a finding we replicated here. It follows that MRI‐based infarct volume has been shown to add predictive information over the NIHSS score in some ischemic stroke subgroups, for instance, cerebellar infarctions [42]. In accordance with this, we found that BD‐tau added the greatest improvement in prognostic accuracy over the NIHSS score and age for posterior ischemic strokes and for the subgroups with infarcts in the brainstem or cerebellum in particular. Of note, BD‐tau alone displayed equivalent prognostic accuracy as the NIHSS score and age in these patient subgroups.

Previous studies on tau in ischemic stroke reported T‐tau concentrations to be increased in the CSF [22, 43]. Further, the presence or absence of serum T‐tau was linked to functional outcome [19, 23] as well as infarct volume [19, 44]. Using the novel highly sensitive BD‐tau assay, we substantiate these results, and we show that plasma BD‐tau and infarct volumes are highly correlated. We previously reported that plasma BD‐tau outperforms NfL as an acute biomarker of outcome for ischemic stroke [29]. This was corroborated in a recent study of large vessel occlusion (LVO) ischemic stroke, where plasma BD‐tau displayed higher discriminative performance for 3‐month functional outcome than GFAP, NfL, and total‐Tau [31]. As this study included LVO patients who were successfully recanalized after endovascular therapy (i.e., thrombectomy) and our study included ischemic stroke patients who were not treated with thrombectomy, the two studies complement each other. We also recently demonstrated that BD‐tau shows no or much weaker correlations with age, renal function, other comorbidities, and self‐identified race/ethnicity, compared with NfL [28], further supporting BD‐tau as a promising brain injury biomarker.

Compared to other complex, common diseases, stroke is lagging in terms of having clinically useful blood‐based biomarkers that accurately reflect the extent of tissue damage caused by the acute injury [15]. For example, plasma concentrations of cardiac troponins (normally present only in cardiomyocytes and released into the circulation upon ischemia) are routinely measured in clinical practice as a complement to clinical symptom assessments and electrocardiograms for the diagnosis of acute coronary syndromes [13, 14]. Analogous to this, plasma concentrations of BD‐tau could have a similar role as an objective indicator of neuronal injury after an ischemic stroke and complement early neuroimaging and clinical symptom assessment. As shown here, BD‐tau could be especially helpful for outcome prediction in mild strokes and posterior circulation ischemic strokes, which are both less likely to be visualized by acute CT and remain a challenge in clinical settings [10, 39]. BD‐tau could be particularly valuable in settings with limited access to neuroimaging or when MRI is not possible due to contraindications. Future studies should address to what extent plasma BD‐tau may assist in acute stroke management, including differential diagnosis (i.e., stroke or stroke mimics), treatment decisions, monitoring, and selection of patients for RCTs.

The principal strength of this study is the inclusion of consecutive hospital‐based ischemic stroke cases that have been extensively characterized and that we used a novel highly specific tau assay that selectively measures tau derived from the brain. Another strength is that in a subgroup of cases, brain infarct volumes and locations were manually determined on MRI scans. In this regard, neuroimaging was relatively late (median 7 days after admission), which has previously been demonstrated to approximate final infarct volume [45]. Limitations to consider include that: (1) For those without MRI, we used the OCSP classification and assessments of symptoms supplemented by neuroimaging findings when possible to classify the cases into different groups based on vascular territory and infarct location. (2) The day of blood sampling was not standardized, nor was the day of investigation with MRI. However, in this regard, correlations between BD‐tau and infarct volume were of similar magnitude in analyses stratified by median day of blood draw or median day of MRI. (3) We used the SSS score and converted this to the NIHSS score in *SAHLSIS*, though this was done with a validated algorithm [37] and has been demonstrated to have comparable performance to the NIHSS for functional outcome prediction [36]. Of note here, a sensitivity analysis based on *SAHLSIS2* had congruent results. (4) The definition of mild stroke varies and we chose NIHSS 0–4 to indicate mild strokes. Other cut‐offs (e.g., NIHSS 0–3, 0–5, and 3–5) may have yielded different results. (5) We acknowledge that there is an overlap between the stroke severity and stroke location analyses, for example, a large proportion of posterior circulation strokes were mild strokes. Given the size of the sample, we could not perform analyses of mild stroke in the different vascular territories separately. (6) We did not assess the predictive accuracy of BD‐tau levels against overall clinical judgment, which is inherently observant dependent and variable between clinicians. In fact, the key advantage of using a biomarker lies in providing objective measures and reducing observant‐dependent biases.

To conclude, analogous to blood‐based cardiac troponins in acute coronary syndrome, BD‐tau shows strong agreement with cerebral infarct volume and adds prognostic value for outcome in acute ischemic stroke. Thus, BD‐tau is an objective, accessible blood‐based biomarker of brain injury that could have clinical utility to complement clinical and neuroimaging evaluation in patients with ischemic stroke to guide individualized treatment. This is especially true for mild ischemic strokes and posterior circulation strokes, where CT neuroimaging assessment is more challenging and for which stroke severity scales are less predictive for outcomes. Future studies in large and independent ischemic stroke cohorts are warranted.

## Author contributions

**Fernando Gonzalez‐Ortiz**: Writing—original draft; methodology. **Lukas Holmegaard**: Writing—original draft; methodology. **Björn Andersson**: Formal analysis; writing—review and editing; visualization. **Cecilia Brännmark**: Writing—review and editing; data curation. **Christian Blomstrand**: Data curation; writing—review and editing. **Henrik Zetterberg**: Writing—review and editing; methodology; funding acquisition. **Katarina Jood**: Writing—review and editing; data curation; methodology; funding acquisition. **Kaj Blennow**: Writing—review and editing; supervision; resources; funding acquisition; conceptualization; methodology. **Christina Jern**: Funding acquisition; writing—review and editing; resources; supervision; conceptualization; methodology. **Tara M. Stanne**: Supervision; funding acquisition; conceptualization; visualization; methodology; writing—original draft; project administration.

## Conflict of interest statement

**K. Blennow** serves as a consultant and on the advisory boards for Acumen, ALZPath, BioArctic, Biogen, Eisai, Julius Clinical, Lilly, Novartis, Ono Pharma, Prothena, Roche Diagnostics, and Siemens Healthineers; on data monitoring committees for Julius Clinical and Novartis; gives lectures, produces educational materials, and participates in educational programs for Biogen, Eisai, and Roche Diagnostics; and is a cofounder of Brain Biomarker Solutions in Gothenburg AB (BBS), which is a part of the GU Ventures Incubator Program, outside the submitted work. **H. Zetterberg** has served at scientific advisory boards and/or as a consultant for Abbvie, Acumen, Alector, Alzinova, ALZPath, Amylyx, Annexon, Apellis, Artery Therapeutics, AZTherapies, Cognito Therapeutics, CogRx, Denali, Eisai, LabCorp, Merry Life, Nervgen, Novo Nordisk, Optoceutics, Passage Bio, Pinteon Therapeutics, Prothena, Red Abbey Labs, reMYND, Roche, Samumed, Siemens Healthineers, Triplet Therapeutics, and Wave, has given lectures in symposia sponsored by Alzecure, Biogen, Cellectricon, Fujirebio, Lilly, Novo Nordisk, and Roche, and is a co‐founder of Brain Biomarker Solutions in Gothenburg AB (BBS), which is a part of the GU Ventures Incubator Program (outside submitted work). **F. Gonzalez‐Ortiz, L. Holmegaard, B. Andersson, C. Brännmark, C. Blomstrand, K. Jood, C. Jern, and T. M. Stanne** report no disclosures relevant to the manuscript.

## Funding information

The Swedish Heart and Lung Foundation (Jern, 20220184); the Swedish Research Council (Jern, 2021‐01114; Blennow, 2022‐00732; Zetterberg, 2023‐00356 and 2022‐01018); Swedish state under the agreement between the Swedish government and the county councils, the ALF agreement (Jern, ALFGBG‐965328; Blennow, ALFGBG‐965240; Jood, ALF GBG‐965417; Zetterberg, ALFGBG‐71320); the King Gustaf V:s and Queen Victoria's Foundation (Jern); the Swedish Alzheimer Foundation (Blennow, AF‐968270), Agneta Prytz‐Folke's and Gösta Folke's Foundation (Jern; Stanne); “Insamlingsstiftelsen” for Neurological Research (Stanne) and the Swedish Stroke Foundation (Stanne).

## Supporting information




Supplementary Material
**Table S1**. Odds ratios (ORs) and 95% confidence intervals (CIs) for 3‐month functional outcome measured by the modified Rankin Scale in the combined *SAHLSIS* and *SAHLSIS2* cohort stratified based on stratified based on clinical stroke severity (i.e. NIHSS < 5 = mild stroke vs NIHSS ≥ 5 = more severe stroke), OCSP Classification (i.e. vascular territory), and CT‐and symptoms‐based anatomical location.
**Figure S1**. Box plots of plasma BD‐tau concentrations in the *SAHLSIS* MRI subgroup stratified by (A) median day of blood sampling or (B) median day of MRI of the brain after index stroke. (C) Scatter plots of BD‐tau versus infarct volumes stratified by day of blood sampling and day of MRI of the brain.
**Figure S2**. Box plots of plasma BD‐tau concentrations per day of blood draw in (A) *SAHLSIS*, (B) *SAHLSIS2* or (C) the combined cohort.
**Figure S3**. Box plots of plasma BD‐tau concentrations in participants with first‐ever stroke versus recurrent stroke in (A) *SAHLSIS*, (B) *SAHLSIS2* or (C) the combined cohort.
**Figure S4**. Box plots of acute‐phase plasma BD‐tau concentrations in ischemic stroke cases with favorable (mRS 0–2) versus unfavorable (mRS 3–6) outcome in the combined cohort stratified by (A) mild stroke (NIHSS < 5) versus (B) more severe stroke (NIHSS ≥ 5).
