# Supplementary material for: Plasma brain‐derived tau correlates with cerebral infarct volume
Source: J Intern Med. 2024 Dec 5;297(2):173–85. doi: 10.1111/joim.20041 (PMC11771704; doi:10.1111/joim.20041)
Supplement: Supplementary file 1 — Supplementary Material Table S1. Odds ratios (ORs) and 95% confidence intervals (CIs) for 3‐month functional outcome measured by the modified Rankin Scale in the combined SAHLSIS and SAHLSIS2 cohort stratified based on stratified based on clinical stroke severity (i.e. NIHSS < 5 = mild stroke vs NIHSS ≥ 5 = more severe stroke), OCSP Classification (i.e. vascular territory), and CT‐and symptoms‐based anatomical location. Figure S1. Box plots of plasma BD‐tau concentrations in the SAHLSIS MRI subgroup stratified by (A) median day of blood sampling or (B) median day of MRI of the brain after index stroke. (C) Scatter plots of BD‐tau versus infarct volumes stratified by day of blood sampling and day of MRI of the brain. Figure S2. Box plots of plasma BD‐tau concentrations per day of blood draw in (A) SAHLSIS, (B) SAHLSIS2 or (C) the combined cohort. Figure S3. Box plots of plasma BD‐tau concentrations in participants with first‐ever stroke versus recurrent stroke in (A) SAHLSIS, (B) SAHLSIS2 or (C) the combined cohort. Figure S4. Box plots of acute‐phase plasma BD‐tau concentrations in ischemic stroke cases with favorable (mRS 0–2) versus unfavorable (mRS 3–6) outcome in the combined cohort stratified by (A) mild stroke (NIHSS < 5) versus (B) more severe stroke (NIHSS ≥ 5). [file JOIM-297-173-s001.pdf]

Supplementary Material

Plasma brain-derived tau correlates with cerebral infarct volume

Fernando Gonzalez-Ortiz, MD, MSc*[1,2]; Lukas Holmegaard, MD, MSc*[3,4]; Björn Andersson, PhD[5]; Cecilia Brännmark, MD, PhD[6,7]; Christian Blomstrand, MD, PhD[3]; Henrik Zetterberg, PhD, MD[1,2,8,9,10,11]; Katarina Jood, MD, PhD[3,4]; Kaj Blennow, MD, PhD‡[1,2,12,13]; Christina Jern, MD, PhD‡[6,14]; Tara M. Stanne PhD‡[6,14]

*These authors contributed equally to this work
‡These authors jointly supervised this work

Author affiliations:

1. Institute of Neuroscience and Physiology, Department of Psychiatry and Neurochemistry, the Sahlgrenska Academy at the University of Gothenburg, Mölndal, Sweden
2. Clinical Neurochemistry Laboratory, Sahlgrenska University Hospital, Mölndal, Sweden
3. Institute of Neuroscience and Physiology, Department of Clinical Neuroscience, the Sahlgrenska Academy, University of Gothenburg, Gothenburg, Sweden
4. Region Västra Götaland, Sahlgrenska University Hospital, Department of Neurology, Gothenburg, Sweden
5. Bioinformatics and Data Center, the Sahlgrenska Academy, University of Gothenburg, Gothenburg, Sweden
6. Institute of Biomedicine, Department of Laboratory Medicine, the Sahlgrenska Academy, University of Gothenburg, Gothenburg, Sweden
7. Region Västra Götaland, Sahlgrenska University Hospital, Department of Medicine Geriatrics and Emergency Medicine, Östra Hospital, Gothenburg, Sweden
8. Department of Neurodegenerative Disease, UCL Institute of Neurology, Queen Square, London, UK
9. UK Dementia Research Institute at UCL, London, UK
10. Hong Kong Center for Neurodegenerative Diseases, Clear Water Bay, Hong Kong, China
11. Wisconsin Alzheimer's Disease Research Center, University of Wisconsin School of Medicine and Public Health, University of Wisconsin-Madison, Madison, WI, USA
12. Paris Brain Institute, ICM, Pitié-Salpêtrière Hospital, Sorbonne University, Paris, France
13. Neurodegenerative Disorder Research Center, Division of Life Sciences and Medicine, and Department of Neurology, Institute on Aging and Brain Disorders, University of Science and Technology of China and First Affiliated Hospital of USTC, Hefei, P.R. China
14. Region Västra Götaland, Sahlgrenska University Hospital, Department of Clinical Genetics and Genomics, Gothenburg, Sweden

Correspondence to: Tara Stanne, PhD, Assoc. Prof.
Institute of Biomedicine, the Sahlgrenska Academy, University of Gothenburg, Box 445, SE-405 30 Gothenburg, Sweden.
E-mail: tara.stanne@gu.se

1. Supplementary Methods

1.1. The Sahlgrenska Academy Study on Ischemic Stroke (SAHLSIS)

1.1.1. Study design

The Sahlgrenska Academy Study on Ischemic Stroke (*SAHLSIS*) is an observational, longitudinal case-control study. Between 1998 and 2003, 600 consecutive cases presenting with with first-ever or recurrent acute ischemic stroke were recruited at 4 stroke units in Western Sweden (two stroke units at the Sahlgrenska University Hospital, one unit at the Södra Älvsborg's Hospital and one unit at Skaraborg's Hospital) as described (Jood et al, 2005)[1]. In brief, the inclusion criteria were as follows; acute onset of symptoms suggestive of stroke, no hemorrhage on neuroimaging, and 18-69 years of age. Exclusion criteria were evaluation showed another etiology of the presenting symptoms than stroke, a diagnosis of cancer at advanced stage, infectious hepatitis or human immunodeficiency virus, and non-caucasian ethnicity. All patients underwent imaging of the brain (computed tomography (CT) and/or magnetic resonance (MR) imaging) as part of the clinical routine investigation, and additional work-up was performed according to national guidelines. It is of note that, as this study included stroke cases aged 18-69 years, the proportion of cases that had imaging of vessels other than carotid doppler ultrasound was relatively high compared to standard clinical routine at the time of inclusion, and 35% additionally underwent magnetic resonance cerebral angiography, 19% transcranial doppler ultrasound, and 11% conventional cerebral angiography. Stroke severity, was measured using the Scandinavian Stroke Scale (SSS) which consists of nine items; consciousness, orientation, eye movement, speech, facial palsy, arm motor power, hand motor power, leg motor power, and gait (Scandinavian Stroke Score Group, 1985).[2]

1.1.2. Vascular risk factors and functional outcome

Information regarding vascular risk factors was registered at inclusion for controls and at a 3-month follow-up visit for cases by examinations and a structured questionnaire, as described (Jood et al, 2005).[1] In brief, hypertension was defined as pharmacological treatment for hypertension and/or systolic blood pressure ≥160 mm Hg, and/or diastolic blood pressure ≥90 mm Hg. Diabetes mellitus was defined as dietary or pharmacological treatment and/or fasting plasma glucose ≥7.0 mmol/L or fasting blood glucose ≥6.1 mmol/L. Smoking habit was coded as current versus never or former (smoking cessation at least one year before inclusion in the study). The modified Rankin Scale (mRS) score was used to assess functional outcome 3 months post-stroke at an in person follow-up. The score was dichotomized into favorable (mRS 0-2) versus unfavorable (mRS 3-6). In total n=3 individuals died during follow-up (i.e. had a mRS score of 6).

1.1.3. Blood sampling

Blood samples were drawn in the acute phase at the 4 stroke units and the same protocol for sample collection and handling was used throughout the study period. Blood sampling was performed within 10 days (median 4 days, interquartile range [IQR] 3-6) of the index stroke event. Venous blood was collected in tubes containing 10% by volume ethylene-diamine-tetra-acetic acid (EDTA, Vacuette, Greiner Bio-One, Essen, Germany) between 8:30 and 10:30 AM after an overnight fast. Plasma was isolated within 2 hours by centrifugation 2000 x *g* at 4 °C for 20 minutes, aliquoted, and stored at -80 °C pending analysis.

1.2. The Sahlgrenska Academy Study on Ischemic Stroke phase 2 (*SAHLSIS2*)

1.2.1. Study design

The ongoing Sahlgrenska Academy Study on Ischemic Stroke phase 2 (*SAHLSIS2*) is an observational, longitudinal case only study initiated in 2015. This study consecutively includes adult first-ever or recurrent stroke patients at two stroke units at the Sahlgrenska University Hospital. In the present study participants with acute ischemic stroke recruited between 2015 and 2020 were included. The inclusion criteria was the same as for *SAHLSIS* except adults of all ages were included. All patients included in this study underwent CT and/or MR imaging of the brain. If clinically indicated, they were also evaluated by CT or MR angiography and perfusion measures. Patients who received recanalization therapy with intravenous thrombolysis (IVT) did so according to national guidelines issued by the Swedish Board of Health and Welfare. Patients receiving endovascular treatment were excluded. Data regarding these treatments were registered at our hospital and reported to national quality registers. The patients also underwent additional work-up according to national guidelines, and they were excluded from this study if further evaluation showed another etiology of the presenting symptoms than ischemic stroke.

1.2.2. Vascular risk factors and functional outcome

In Sweden, all hospitals treating patients with acute stroke report comprehensive data on for instance stroke severity (i.e. National Institutes of Health Stroke Scale (NIHSS) score at admission), demographics, vascular risk factors, and outcomes to the national quality register for stroke, Riksstroke. Data are entered into this register by licensed healthcare personnel at each hospital, and the coverage rate is about 95% (www.riksstroke.org/general-information/). In *SAHLSIS2*, we present data on diabetes mellitus, hypertension, and smoking at baseline reported from our hospital to Riksstroke. We also registered data from a questionnaire developed by Riksstroke on self-reported dependency in specified domains (mobility, dressing, and toileting), living conditions, and need of help or support from next of kin 3 months post-stroke as described (Eriksson et al, 2007).[3] Data from this questionnaire were then used to estimate the modified Rankin Scale (mRS) scores 0-2, 3, 4 or 5 using a validated translation algorithm.[3] It is of note that it is not possible to distinguish mRS 0 from 1 or 2 using this algorithm. The mRS score was dichotomized into favorable (mRS 0-2) versus unfavorable (mRS 3-6). In total n=10 cases died during follow-up (i.e. had a mRS score of 6).

1.2.3. Blood sampling

Blood samples were drawn, and plasma was isolated and stored as described for *SAHLSIS*. The median day of blood draw was 2 [IQR 2-4] after index stroke.

2. Supplementary Results

2.1. MRI subgroup

In exploratory analyses, BD-tau concentrations were not significantly different when stratified by median day of blood draw (< 4 vs ≥ 4 days after index stroke) or median day of MRI (< 7 vs ≥ 7 days after index stroke; t-test *p* > 0.05 for both; Figure S1). Correlations between BD-tau and infarct volume were similar when stratified by day of blood draw (Spearman's *rho* 0.72, *p* <0.001 for both < 4 vs ≥ 4 days after index stroke), or day of MRI (MRI < 7 days: *rho* 0.74; MRI ≥ 7 days: *rho* 0.71; *p* <0.001 both).

2.2. SAHLSIS and SAHLSIS2

BD-tau concentrations were not correlated with day of blood draw in *SAHLSIS* (Spearman's *rho* = 0.070, *p* >0.05) and were weakly correlated with day of blood draw in *SAHLSIS2* (Spearman's *rho* = 0.136, *p* =0.03; Figure S2). BD-tau concentrations were not significantly different in patients with first-ever versus recurrent strokes (t-test *p* > 0.05; Figure S3).

We next evaluated whether BD-tau could differentiate favorable and unfavorable outcome for mild strokes (NIHSS < 5) and more severe strokes (NIHSS ≥ 5). Acute plasma BD-tau concentrations were significantly elevated in cases that experienced an unfavorable outcome compared to favorable outcome in both severity groups (t-test *p* <0.001 both; Figure S4).

Higher levels of BD-tau were associated with an increased odds of unfavorable outcome after adjustment for age and NIHSS score, and the odds ratio per doublilng of BD-tau was highest for mild strokes (NIHSS < 5) compared to more severe stroke (NIHSS ≥ 5); posterior circulation infarcts compared to anterior circulation infarcts; and brainstem/ cerebellar infarcts compared to right or left hemisphere infarcts (Table S1).

Supplementary Tables

Table S1. Odds ratios (ORs) and 95% confidence intervals (CIs) for 3-month functional outcome measured by the modified Rankin Scale in the combined *SAHLSIS* and *SAHLSIS2* cohort stratified based on stratified based on clinical stroke severity (i.e. NIHSS < 5 = mild stroke vs NIHSS ≥ 5 = more severe stroke), OCSP Classification (i.e. vascular territory), and CT-and symptoms-based anatomical location.

Model	Variable	OR (95% CI)	*P*
**Mild stroke (NIHSS < 5)**			
Ln-BD-tau	BD-tau	2.78 (2.05-3.77)	5E-11
NIHSS	NIHSS	1.58 (1.26-1.98)	7E-05
NIHSS + age	NIHSS	1.67 (1.31-2.11)	3E-05
	age	1.08 (1.05-1.12)	3E-08
NIHSS + age + LnBD-tau	NIHSS	1.70 (1.32-2.18)	3E-05
	age	1.06 (1.03-1.09)	1E-04
	BD-tau	2.55 (1.83-3.55)	4E-08
**More severe stroke (NIHSS ≥ 5)**			
Ln-BD-tau	BD-tau	1.86 (1.45-2.38)	8E-07
NIHSS	NIHSS	1.15 (1.09-1.22)	2E-06
NIHSS + age	NIHSS	1.17 (1.10-1.24)	4E-07
	age	1.03 (1.01-1.05)	0.008
NIHSS + age + LnBD-tau	NIHSS	1.16 (1.09-1.23)	5E-06
	age	1.02 (1.00-1.05)	0.048
	BD-tau	1.75 (1.35-2.28)	3E-05
**Anterior circulation (TACI+PACI)**			
Ln-BD-tau	BD-tau	2.07 (1.63-2.63)	2E-09
NIHSS	NIHSS	1.25 (1.18-1.32)	3E-14
NIHSS + age	NIHSS	1.05 (1.03-1.08)	7E-05
	age	0.01 (0.00-0.00)	6E-09
NIHSS + age + LnBD-tau	NIHSS	1.24 (1.17-1.32)	1E-12
	age	1.04 (1.02-1.07)	0.001
	BD-tau	1.63 (1.24-2.13)	4E-04
**Posterior circulation (POCI)**			
Ln-BD-tau	BD-tau	2.53 (1.60-4.02)	8E-05
NIHSS	NIHSS	1.15 (1.05-1.26)	2E-03
NIHSS + age	NIHSS	1.03 (1.00-1.07)	7E-02
	age	0.01 (0.00-0.00)	3E-04
NIHSS + age + LnBD-tau	NIHSS	1.21 (1.09-1.34)	5E-04
	age	1.03 (0.99-1.06)	0.161
	BD-tau	3.02 (1.75-5.21)	7E-05
**Right + left hemisphere**			
Ln-BD-tau	BD-tau	2.30 (1.89-2.80)	8E-17
NIHSS	NIHSS	1.29 (1.23-1.35)	3E-24
NIHSS + age	NIHSS	1.31 (1.25-1.38)	9E-25
	age	1.06 (1.04-1.08)	3E-08
NIHSS + age + LnBD-tau	NIHSS	1.29 (1.22-1.36)	1E-20
	age	1.05 (1.02-1.07)	3E-05
	BD-tau	1.78 (1.41-2.25)	1E-06

**Brainstem + cerebellum**			
Ln-BD-tau	BD-tau	2.61 (1.51-4.53)	0.001
NIHSS	NIHSS	1.17 (1.05-1.30)	0.003
NIHSS + age	NIHSS	1.18 (1.06-1.31)	0.002
	age	1.03 (0.98-1.09)	0.209
NIHSS + age + LnBD-tau	NIHSS	1.31 (1.12-1.52)	0.001
	age	1.03 (0.98-1.08)	0.227
	BD-tau	4.24 (1.93-9.31)	3E-04

3. Supplementary Figures

Figure S1. Box plots of plasma BD-tau concentrations in the *SAHLSIS* MRI subgroup stratified by (A) median day of blood sampling or (B) median day of MRI of the brain after index stroke. (C) Scatter plots of BD-tau versus infarct volumes stratified by day of blood sampling and day of MRI of the brain.

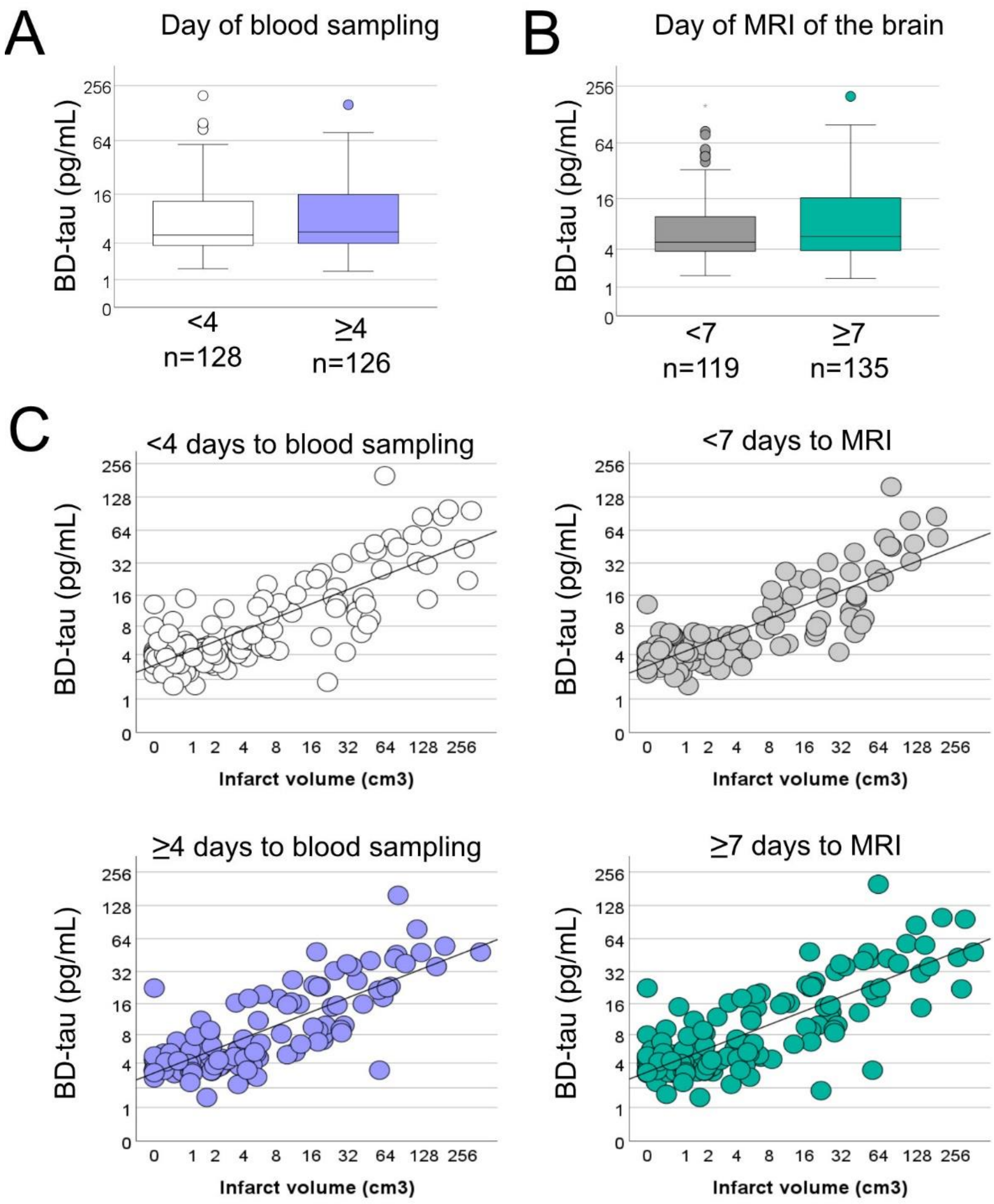

Figure S2. Box plots of plasma BD-tau concentrations per day of blood draw in (A) *SAHLSIS*, (B) *SAHLSIS2* or (C) the combined cohort.

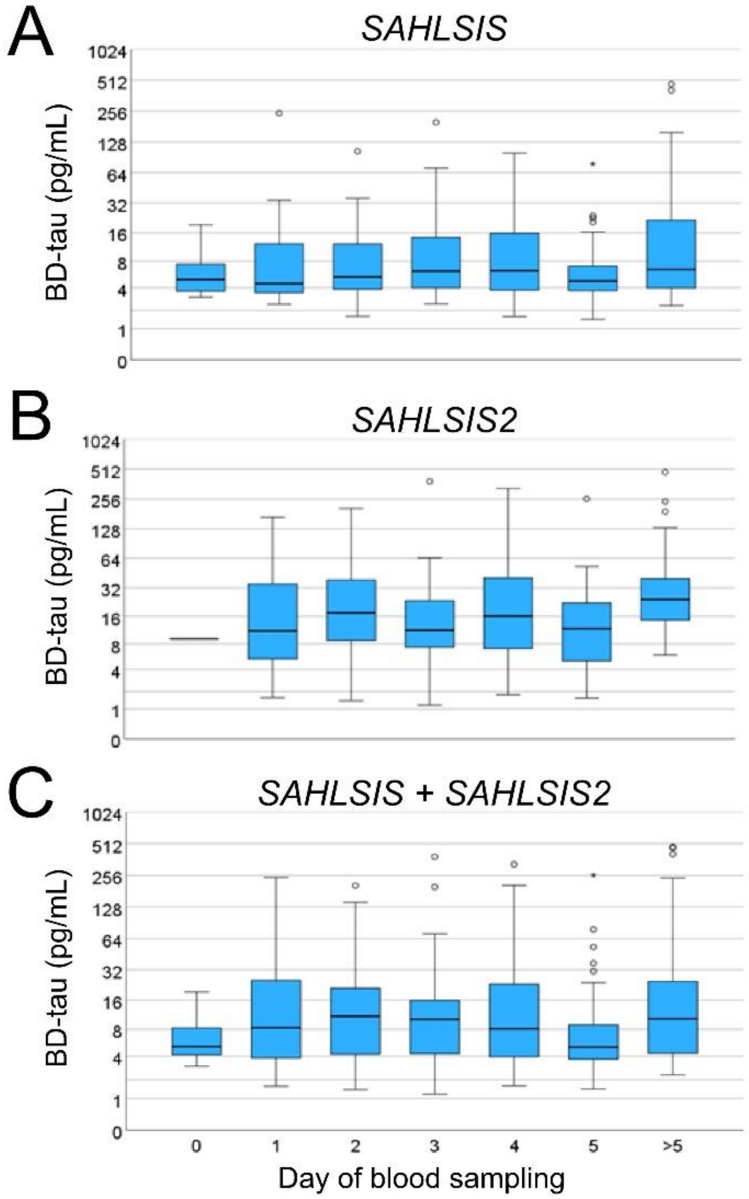

Figure S3. Box plots of plasma BD-tau concentrations in participants with first-ever stroke versus recurrent stroke in (A) *SAHLSIS*, (B) *SAHLSIS2* or (C) the combined cohort.

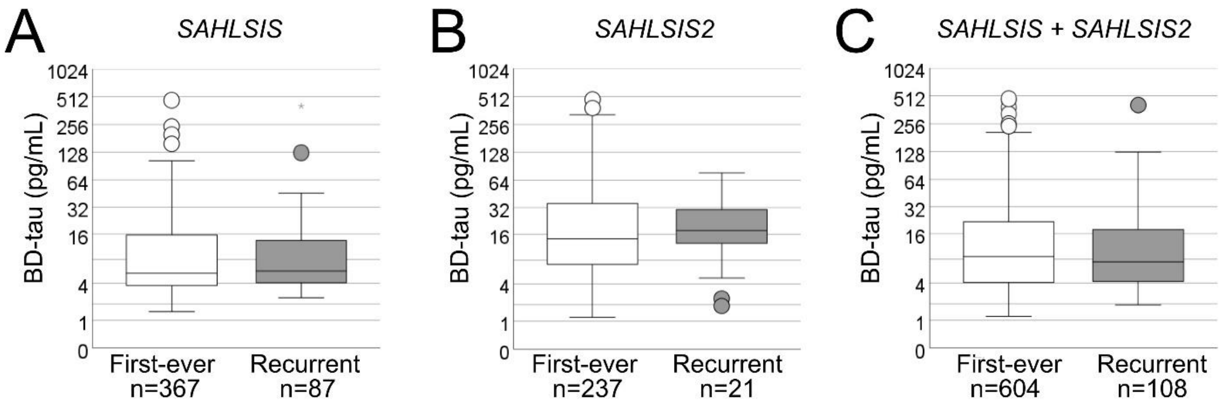

Figure S4. Box plots of acute-phase plasma BD-tau concentrations in ischemic stroke cases with favorable (mRS 0-2) versus unfavorable (mRS 3-6) outcome in the combined cohort stratified by (A) mild stroke (NIHSS < 5) versus (B) more severe stroke (NIHSS ≥ 5). ***Student's t-test p-value < 0.001.

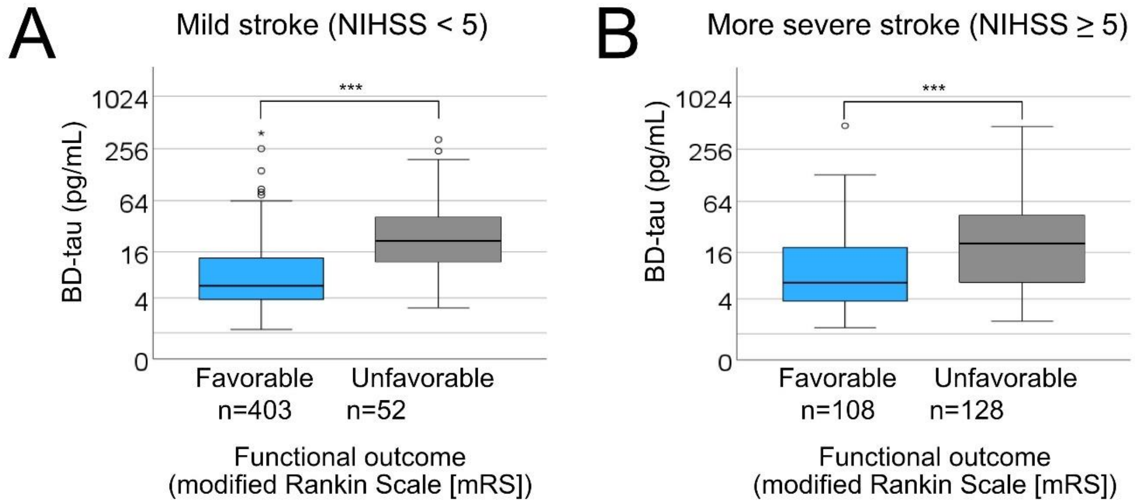

4. Supplementary References

1. Jood K, Ladenvall C, Rosengren A, Blomstrand C, Jern C. Family history in ischemic stroke before 70 years of age: the Sahlgrenska Academy Study on Ischemic Stroke. *Stroke* 2005; **36:** 1383-7.
2. Scandinavian Stroke Study Group: Multicenter trial of hemodilution in ischemic stroke--background and study protocol. *Stroke* 1985; **16:** 885-90.
3. Eriksson M, Appelros P, Norrving B, Terént A, Stegmayr B. Assessment of functional outcome in a national quality register for acute stroke: can simple self-reported items be transformed into the modified Rankin Scale? *Stroke* 2007; **38:** 1384-6.
4. Gonzalez-Ortiz F, Turton M, Kac PR, et al. Brain-derived tau: a novel blood-based biomarker for Alzheimer's disease-type neurodegeneration. *Brain* 2023; **146:** 1152-1165.
